# Rapid profiling of polymyxin B susceptibility in Gram-negative bacteria with a novel polymyxin-derived fluorescent probe

**DOI:** 10.1016/j.jare.2026.01.019

**Published:** 2026-01-10

**Authors:** Bing Zhang, Wanida Phetsang, Sanjaya Kc, Mark S. Butler, Ruby Pelingon, Alysha G. Elliott, Maite Amado, Alejandra Gallardo-Godoy, Matthew A. Cooper, Mark A.T. Blaskovich

**Affiliations:** a Centre for Superbug Solutions, Institute for Molecular Bioscience, The University of Queensland, Brisbane, Queensland, Australia; b School of Chemistry and Molecular Biosciences, The University of Queensland, Brisbane, Queensland, Australia^1^; c Biointelect Pty Ltd, Brisbane, Queensland, Australia^1^; d Sitala Bio Ltd, Unit D6, Grain House, Cambridge CB22 5LD, UK^1^

**Keywords:** Antimicrobial resistance, Fluorescent probe, Polymyxin B, Flow cytometry

## Abstract

**Introduction:**

Polymyxins are considered last-resort antibiotics for treating Gram-negative bacterial infections. The alarming global rise in polymyxin-resistant bacteria coupled with the slowness of conventional antimicrobial susceptibility testing methods leads to an unmet need for rapid susceptibility testing to facilitate timely and targeted therapeutic interventions.

**Objectives:**

In this study, we aimed to develop novel polymyxin-derived fluorescent probes and improve the identification of polymyxin resistance by using these pathway-specific tools.

**Methods:**

We designed, synthesized, and profiled a polymyxin-fluorophore derivative (PMX-NBD) with the small fluorophore NBD attached the central cyclic core of polymyxin via a triazole linker. We then applied PMX-NBD to develop RAPID_FC_, a flow cytometry method that can rapidly and accurately profile polymyxin susceptibility of various Gram-negative pathogens.

**Results:**

In contrast to many previously reported polymyxin probes, the fluorescent PMX-NBD retains the antimicrobial activity and profile of the parent polymyxin. Furthermore, we tested probe labeling in 49 strains encompassing *E. coli*, *K. pneumoniae*, *P. aeruginosa*, *A. baumannii* and *S. aureus* with a range of polymyxin sensitivity, leading to a susceptibility testing method RAPID_FC_. Susceptibility to polymyxin B was defined by the difference between the Geometric Mean of PMX-NBD fluorescence intensity at the two concentrations, with sensitive strains showing a greater variation compared to resistant strains. The RAPID_FC_ method is easy to implement and only requires 45 min compared to the broth microdilution method, which requires at least 24 h. However, the standard protocol failed to provide clear discrimination for *A. baumannii*, a key WHO-priority pathogen, necessitating a modified, species-specific approach for this organism.

**Conclusions:**

The RAPID_FC_ method, based on the polymyxin-derived fluorescent probe, is fast and applicable to multiple species, making it a promising antimicrobial susceptibility method to guide appropriate therapy in the clinic. The case of *A. baumannii* demonstrates that species-specific resistance mechanisms may require tailored optimization of detection protocols.

## Highlights

• We designed and synthesized a fluorescent polymyxin probe, PMX-NBD, via ‘click’ chemistry.
• The modified derivatives retained similar antimicrobial activity to the parent polymyxin B antibiotic.
• PMX-NBD showed its ability to label Gram-negative bacteria.
• A flow-cytometry method was developed to quickly determine bacterial susceptibility to polymyxin B within 45 min.
• It is a promising method to determine the resistance profile of bacterial isolates.

## Graphical abstract

**Figure:**
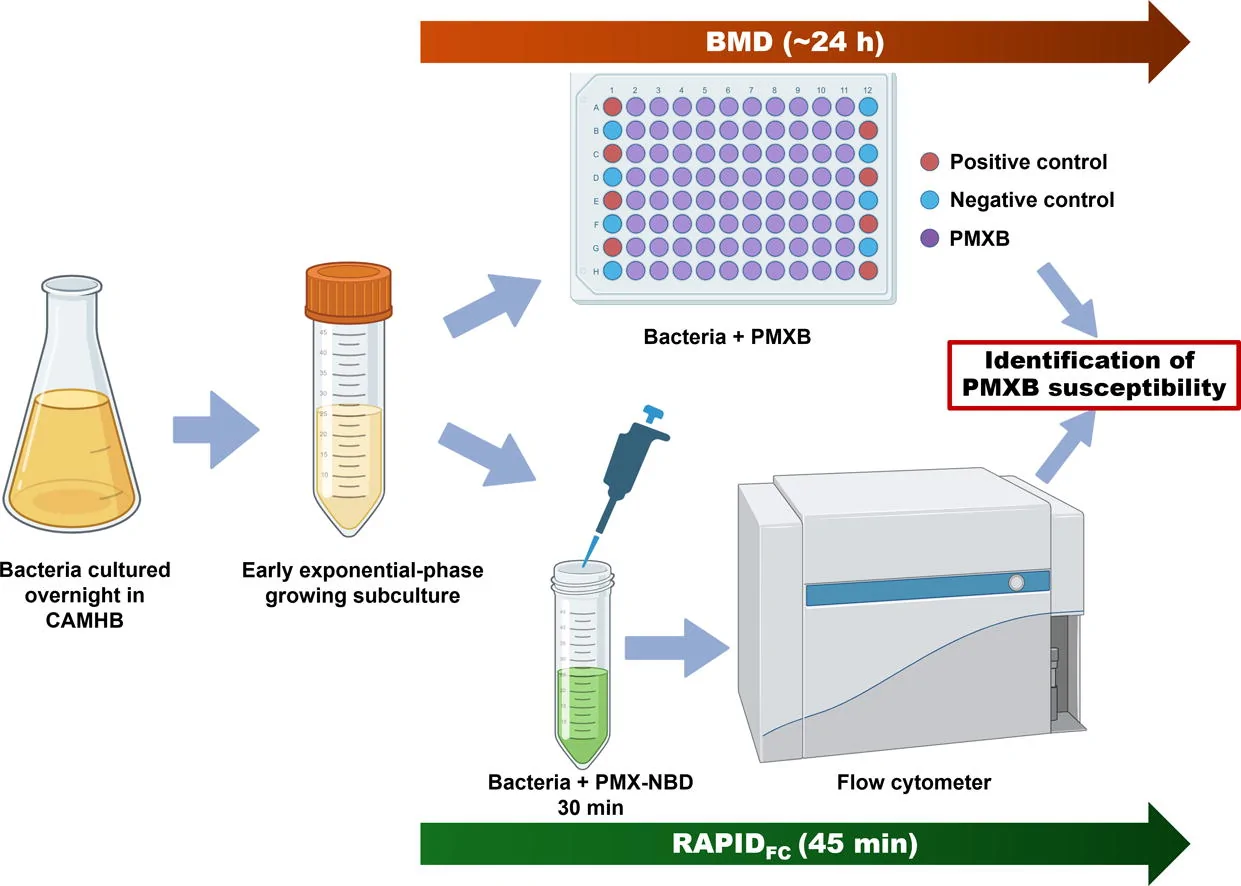

## Introduction

The pentabasic lipo-decapeptide polymyxin class of antibiotics were first discovered in *Bacillus polymyxa* in 1947 [1] and include a number of closely related complexes (A, B, C, D, E) [2]. Of these, only polymyxin B (PMXB, Fig. 1) and polymyxin E (colistin – generally administered as the prodrug colistin methanesulfonate, known as colistimethate) are produced commercially for clinical use [3], [4]. Three polymyxin analogs are commercially available worldwide: colistin methanesulfonate for injection, polymyxin B sulfate for injection and colistin sulfate. In recent clinical practice, applications including intracerebroventricular, intrathecal administration and the nebulized inhalation method, have gained broader acceptance [5], [6].

**Fig. 1:**
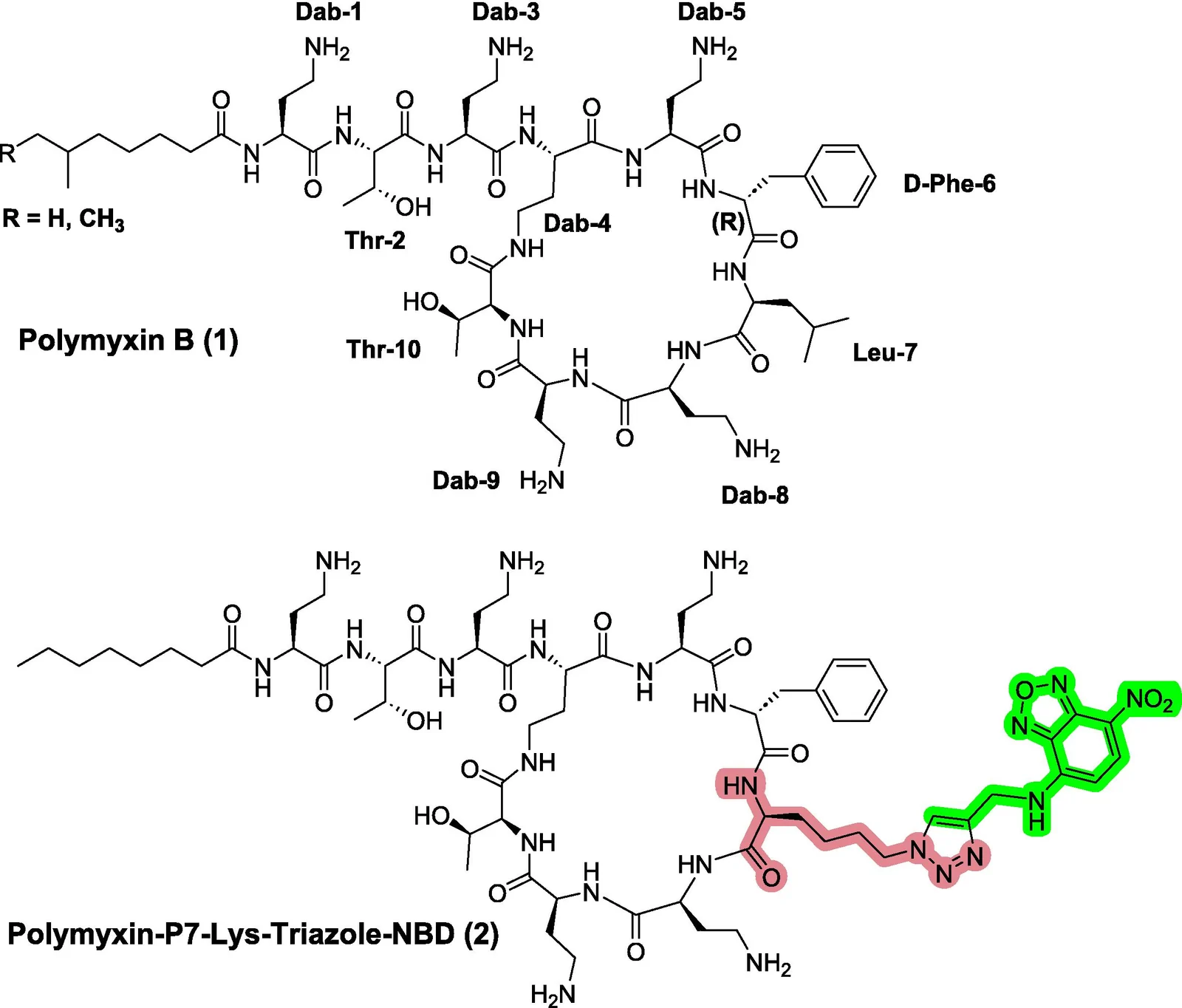
**Chemical structures of polymyxin B and polymyxin B fluorescent probe**.

Polymyxins have been employed in clinical practice for over six decades. Given their activity against numerous Gram-negative bacilli and coccobacilli, polymyxins are considered as the last-resort antibiotics for the treatment of multidrug-resistant (MDR) and/or extensively drug-resistant (XDR) Gram-negative infections [7]. Unfortunately, many bacteria, including the four major superbugs of greatest concern, *Escherichia coli*, *Klebsiella pneumoniae*, *Pseudomonas aeruginosa*, and *Acinetobacter baumannii*, have developed resistance to polymyxins [8]. Epidemiologically, polymyxin resistance demonstrates a concerning global increase, with notably high and variable rates in key pathogens, particularly among carbapenem-resistant strains. The situation is exacerbated by the plasmid-mediated, horizontally transferable *mcr-1* gene, which has become widely disseminated in animal reservoirs and the environment, posing a significant threat to public health by facilitating resistance spread to human pathogens [9], [10], [11], [12], [13], [14], [15]. Considering the rising use of polymyxins in clinical practice and new analogs in clinical development, it is of considerable interest to rapidly determine the resistance profile of isolates [8], [16], [17].

Traditional antimicrobial susceptibility testing methods for determining polymyxin resistance have a variety of limitations, e.g. broth microdilution (BMD) assays need at least 24 h after an overnight inoculation, agar-based methods, such as disk diffusion and Etest, can be affected by the poor diffusion of the antibiotics, and the VITEK (bioMérieux) is considered unreliable for determining polymyxin resistance [18], [19], [20]. Simple phenotypic ‘agar spot’ and ‘agar drop’ tests were recently found to give good correlation to BMD assays for colistin, but not PMXB [21]. A limited number of novel methods have been developed for rapid antimicrobial susceptibility testing [22], including a bacterial cytological profiling method applied to *Staphylococcus aureus* [23] and an acoustic flow cytometry approach designed for the determination of carbapenem susceptibility [24]. A colistin-specific assay uses flow cytometry to measure bacterial fluorescent uptake after treatment with various concentrations of colistin [25], [26]. While not specified in the research articles, the fluorochrome used is presumably a non-specific dye as the patent application states ‘preferably acridine dye, phenanthridine dye, rhodamine dye, cyanine dye, oxazine dye, or a fluorone dye’ [27]. Based on these studies using various non-selective fluorescent probes, we believe that there is potential to improve the identification of polymyxin resistance by using pathway-specific antibiotic-derived fluorescent probes developed within our group.

Fluorescent probes are molecules that can be used to track the localization of fluorescently labeled molecules, or which alter their fluorescence emission in reaction to a binding event, chemical reaction, or alterations in their surrounding environment. Fluorescent probes are extensively utilized to study bacteria [28], but only a few antibiotic-derived fluorescent probes have been reported to date [29], with applications including cellular imaging [30], [31], [32], [33], [34], [35] and mode of action studies [36], [37], [38], [39], [40]. It is worth noting that among these probes, many do not report if they have retained the antimicrobial activity of their parent antibiotics, which raises doubts about how accurately they represent the progenitor’s characteristics. Polymyxin-derived fluorescent probes have previously been reported but are either prepared by non-specific derivatization of one or more of the diaminobutyric acid (Dab) residue free amino groups that are essential for polymyxin activity, or by modification of the exocyclic ‘tail’. The earliest dansyl-derivatives non-selectively derivatized the multiple free amine groups on the five Dab, producing mixtures of mono-, di-, tri-, and tetra-substituted compounds with reduced antibacterial activity [41], [42], [43], [44]. Commercial Oregon Green- and BODIPY-polymyxin analogs (LifeSciences/Invitrogen/ThermoFisher, Cat No. P13236& P13235; now discontinued) were similarly non-selectively derivatized [45], [46], [47]. A Cy3 analog was prepared in 2018 in a similar manner; activity was not reported [32]. Several specifically synthesized dansyl-derivatives have modified the exocyclic ‘tail’, with varying effects on activity [44], [48], [49], [50].

Our laboratory has successfully designed and synthesized a fluorescent polymyxin-fluorophore derivative that uniquely attaches the small fluorophore 7-**n**itro**b**enz-2-oxa-1,3-**d**iazol-4-yl (NBD, green) to the central cyclic core of polymyxin via a triazole linker generated by ‘click’ chemistry between an azidolysine residue at the P7 position of PMXB and an alkyne substituent on the NBD fluorophore. The resulting adduct, polymyxin-P7-Lys-Tz-NBD (PMX-NBD **2**, Fig. 1), retains the antimicrobial activity and profile of the parent polymyxin, in contrast to most of the previously reported polymyxin probes. Here, we apply PMX-NBD to develop RAPID_FC_, a flow cytometry method that can rapidly and accurately profile polymyxin susceptibility of various Gram-negative pathogens including *E. coli*, *K. pneumoniae*, and *P. aeruginosa*. We tested 49 strains encompassing *E. coli*, *K. pneumoniae*, *P. aeruginosa*, *A. baumannii* and *S. aureus* with a range of polymyxin sensitivity (Table 2). These bacteria were incubated with PMX-NBD at two concentrations (1 μg/mL and 16 μg/mL) for 30 min, followed by the measurement of fluorescence intensity using flow cytometry. Susceptibility to PMXB was defined by careful calculation of the difference between the Geometric Mean (GMean) of PMX-NBD fluorescence intensity at the two concentrations, with sensitive strains showing a greater variation compared to resistant strains for *E. coli*, *K. pneumoniae* and *P. aeruginosa*.

**Table 1:** Antibiotic activity of various probes against a panel of bacteria.

	MIC [μg/mL]*										
Compounds	*E. coli*	*K. pneumoniae*				*P. aeruginosa*			*A. baumannii*		*S. aureus*
	ATCC 25922	ATCC 700603	ATCC BAA-2146	Greek CI	Greek CI	ATCC 27853	FADDI-PA070	PA9704-Denmark	ATCC 19606	Greek CI	ATCC 25923
	FDA Control	MDR	NDM-1	PMXB Resistant	PMXB Resistant	Control strain	PMXB Resistant	PMXB Resistant	Type	PMXB Resistant	MSSA
Polymyxin B (PMXB)	0.25–0.5	0.25–0.5	0.25–0.5	32, 64	32, 64	0.5–2	16, 32	> 64	0.25, 1	64	> 64
PMX-P7-N3	0.063–0.5	0.25–1	0.125–0.5	> 64	32, 64	0.25–0.5	> 64	> 64	0.063–0.125	64	> 64
PMX-P7-Tz-NBD (PMX-NBD)	0.25–0.5	0.5–1	1	> 64	> 64	0.5	> 64	> 64	4	≥ 64	> 64

*Range obtained from of a minimum of 3 biological replicates in duplicate.

**Table 2:** Susceptibility to PMXB of the tested strains.

Species	Strain ID	Description	MIC_BMD_ (μg/mL)	BMD	RAPID_FC_
*E. coli*					
GN_284	AR Bank #0119	ESBL, NDM-1	0.25	S^a^	S^a^
GN_112	ATCC 700926	K12	0.25	S^a^	S^a^
GN_315	AR Bank #0150	NDM-5	0.25	S^a^	S^a^
GN_293	AR Bank #0128	NDM-1	0.25	S^a^	S^a^
GN_314	AR Bank #0149	NDM-7	0.25	S^a^	S^a^
GN_283	AR Bank #0118	NDM-1	0.25	S^a^	S^a^
GN_001	ATCC 25922	FDA strain, Seattle 1946	0.25, 0.5	S^a^	S^a^
GN_377	AR Bank #0494	MDR, *mcr-1*	4	NS^b^	NS^b^
GN_371	AR Bank #0346	*mcr-1*	4	NS^b^	NS^b^
GN_378	AR Bank #0495	*mcr-1*	4	NS^b^	NS^b^
GN_376	AR Bank #0493	*mcr-1*	4	NS^b^	NS^b^
GN_375	AR Bank #0350	*mcr-1*	4	NS^b^	NS^b^
GN_381	AR Bank #0538	*mcr-2*	4	NS^b^	NS^b^

*K. pneumoniae*					
GN_294	AR Bank #0129	KPC-3, ESBL	0.25	S^a^	S^a^
GN_045	ATCC BAA-2146	NDM-1	0.25, 0.5	S^a^	S^a^
GN_131	CI^c^ 100575214:01	XDR, KPC-2, ESBL	0.25	S^a^	S^a^
GN_306	AR Bank #0141	OmpK35	0.5, 1	S^a^	S^a^
GN_291	AR Bank #0126	OmpK36	0.25, 1	S^a^	S^a^
GN_303	AR Bank #0138	NDM-7	0.25, 0.5	S^a^	S^a^
GN_300	AR Bank #0135	OmpK35	0.25	S^a^	S^a^
GN_003	ATCC 700603	ESBL	0.25, 0.5	S^a^	S^a^
GN_290	AR Bank #0125	OmpK35	16, 32	NS^b^	NS^b^
GN_106	CI^c^ 138-16357-20362	XDR, KPC-2, ESBL	32, 64	NS^b^	NS^b^
GN_102	CI^c^ 100650661:1	XDR, KPC-2, ESBL	32, 64	NS^b^	NS^b^
GN_107	CI^c^ 138-16627-1129	XDR, KPC-2, ESBL	> 64	NS^b^	NS^b^
GN_183	Induced Serial passage	XDR, PMXB resistant	≥ 256	NS^b^	NS^b^

*P. aeruginosa*					
GN_042	ATCC 27853	Control strain, OXA-50, PAO	0.5–2	S^a^	S^a^
GN_262	CI^c^		0.5	S^a^	S^a^
GN_272	NCTC 13437	ESBL, VEB	0.5, 1	S^a^	S^a^
GN_043	FADDI-PA070	OXA-50, PAO	16, 32	NS^b^	NS^b^
GN_177	FADDI-PA067	Clinical isolate, resistant	8	NS^b^	NS^b^
GN_105	PA9704	Clinical isolate, resistant	> 64	NS^b^	NS^b^
GN_1225	Pa-1225-NG	Clinical isolate	0.25	S^a^	S^a^
GN_960	Pa-0960-TN	Clinical isolate	2	S^a^	S^a^
GN_1239	Pa-1239-NG	Clinical isolate	2	S^a^	S^a^
GN_964	Pa-0964-KE	Clinical isolate	8	NS^b^	NS^b^

*S. aureus*					
GP_001	ATCC 25923	Seattle 1945	> 64	NS^b^	NS^b^

*A. baumannii*					
GN_034	ATCC 19606	Type strain	0.25, 1	S^a^	–
GN_335	AR Bank #0278	ESBL	0.25, 0.5	S^a^	–
GN_093	CI^c^ Ptyela 100734512:2	XDR, ESBL, PMXB resistant	64	NS^b^	–
GN_095	CI^c^ 100603857:1	METALLO-OR OXA, resistant to polypeptides	64	NS^b^	–
GN_653	Ab-0653-BR	Clinical isolate	0.5	S^a^	–
GN_851	Ab-0851-GE	Clinical isolate	0.5	S^a^	–
GN_858	Ab-0858-MA	Clinical isolate	0.25	S^a^	–
GN_624	Ab-0624-BR	Clinical isolate	4	NS^b^	–
GN_636	Ab-0636-BR	Clinical isolate	4	NS^b^	–
GN_633	Ab-0633-BR	Clinical isolate	> 32	NS^b^	–
GN_834	Ab-0834-BR	Clinical isolate	> 32	NS^b^	–
GN_860	Ab-0860-JO	Clinical isolate	> 32	NS^b^	–

a Susceptible to PMXB, MIC ≤ 2 μg/mL.

b Non-susceptible to PMXB, MIC ≥ 4 μg/mL.

c Clinical isolate.

## Materials and methods

For chemical synthesis and characterization of PMX-NBD, refer to the **Supplementary Materials**.

**Bacterial isolates and chemicals.** The strains used in this study were obtained from the American Type Culture Collection (ATCC), the CDC AR Isolate Bank collection and clinical collaborators, or generated by resistance-selection studies within our group [51] (strain profiles provided in Table 2). Bacteria were grown in Cation-Adjusted Mueller Hinton Broth (CAMHB, Bacto Laboratories, Cat No. 212322). PMX-NBD was initially dissolved in sterile double-distilled water to achieve a stock concentration of 1.28 mg/mL and diluted in the culture medium to different working concentrations.

**Inhibition of microbial growth.** Minimal inhibitory concentrations (MICs) were determined by the Clinical and Laboratory Standards Institute (CLSI) broth microdilution method, the gold standard for antimicrobial susceptibility testing [52]. The overnight bacterial culture was diluted 40-fold in fresh CAMHB and incubated at 37 °C for 1.5–2 h (OD_600nm_ = 0.4–0.6). Then the compound, dissolved in sterile milli-Q water, was serially diluted two-fold across the wells of 96-well polystyrene flat bottom plates (Corning, Cat No. 3370), with concentrations ranging from 0.063 μg/mL to 128 μg/mL, plated in duplicate. The resultant mid-log phase culture was diluted to a concentration of 1 × 10^6^ CFU/mL. After that, 50 μL was added to each well of the compound-containing 96-well plates, achieving a cell density of 5 × 10^5^ CFU/mL, and a final compound concentration range of 0.031 μg/mL to 64 μg/mL. Subsequently, the plates were incubated at 37 °C for 18–24 h. MICs were determined by visual inspection, being defined as the lowest concentration at which no visible growth occurred. Antimicrobial susceptibility testing breakpoints were interpreted according to the CLSI M100 guidelines, 33rd edition [52].

**Live cell confocal microscope fluorescence imaging of bacteria with PMX-NBD.** A sample of each *E. coli* overnight culture was diluted 40-fold in CAMHB medium and then incubated at 37 °C for 1.5–2 h to produce exponential phase cells (OD_600nm_ = 0.4–0.6). Subsequently, 3 mL of the obtained mid-log phase cultures were collected and centrifuged in an Eppendorf tube and washed once with HBSS (3, 500 × *g*, for 2 min). Following this, 1 mL of PMX-NBD solution at the desired concentration in HBSS was introduced and incubated for 30 min at 37 °C with shaking. After washing once with HBSS, bacteria were stained with Hoechst 33342 (20 μg/mL, 10 min on ice) and then washed once with HBSS after staining. Next, bacteria were stained with FM4-64FX (5 μg/mL, 5 min on ice), with an additional HBSS wash after staining. Finally, the bacterial pellets were resuspended in HBSS and mounted on an agarose pad, ready for high-resolution confocal imaging analysis using an Inverted LSM 880 Fast Airyscan (objective lens: 63×/1.40 OIL).

**Flow cytometry susceptibility test of Gram-negative bacteria labeled with the fluorescent probe PMX-NBD.** A sample of each bacterial overnight culture was diluted 40-fold in fresh CAMHB and incubated at 37 °C for 1.5–2 h (OD_600nm_ = 0.4–0.6). Subsequently, 1 mL of the obtained mid-log phase cultures were collected into an Eppendorf tube and washed once with HBSS (3, 500 × *g*, for 2 min), then a solution (1 mL) of PMX-NBD (1, 16 μg/mL) was introduced to the Eppendorf tube containing the bacteria pellet, and shaken for 30 min at 37 °C. Bacterial cell pellets were collected by centrifugation and washed again with HBSS (3, 500 × *g*, for 2 min) and then the resulting pellets were resuspended and diluted in HBSS for analysis using a Cytoflex S flow cytometer (Beckman Coulter, Inc. 250 S. Kraemer Blvd. Brea, CA 92821 U.S.A.). **Flow cytometry settings for *E. coli* and *K. pneumoniae*:** Forward Scattering: Gain of 97; Side Scattering: Gain of 200; FITC channel (Excitation 488 nm/Emission 525/20 nm): Gain of 167; Manual thresholds for Side Scatter in 2, 000. **Flow cytometry settings for *P. aeruginosa*:** Forward Scattering: Gain of 200; Side Scattering: Gain of 886; FITC channel (Excitation 488 nm/Emission 525/20 nm): Gain of 167; Manual thresholds for Side Scatter in 5, 500. **Flow cytometry settings for *A. baumannii*:** Forward Scattering: Gain of 97; Side Scattering: Gain of 500; FITC channel (Excitation 488 nm/Emission 525/20 nm): Gain of 167; Manual thresholds for Side Scatter in 3, 000. **Flow cytometry settings for *S. aureus*:** Forward Scattering: Gain of 50; Side Scattering: Gain of 300; FITC channel (Excitation 488 nm/Emission 525/20 nm): Gain of 167; Manual thresholds for Side Scatter in 3, 000. For all samples, 40, 000 events were collected.

**Statistical analysis.** Flow cytometer collected data were analyzed in Kaluza Analysis 2.1 (Beckman Coulter, Inc. 250 S. Kraemer Blvd. Brea, CA 92821 U.S.A.) by a single user. Statistical software (GraphPad Prism 8, San Diego, CA, U.S.A.) was used to analyze categorization results.

## Results and Discussion

### Design and synthesis of PMXB-linked fluorophores

We have previously conjugated three small fluorophores to several different classes of antibiotics, using 7-(dimethylamino)-coumarin-4-acetic acid (DMACA: absorbs in the violet and emits blue fluorescence), 7-nitrobenz-2-oxa-1,3-diazol-4-yl (NBD: absorbs in the blue and emits green fluorescence), and dansyl (DNS, dimethylamino-sulfonyl: absorbs in the violet and emits blue → green fluorescence). NBD is the smallest fluorophore, whereas DMACA and DNS possess relatively similar molecular sizes. To date, these have been linked to linezolid [53], trimethoprim [54], roxithromycin [55] ciprofloxacin [39] and vancomycin [56], with the probes used to visualize antibiotic localization and develop assays for antibiotic efflux and outer membrane permeabilization. For this project, to allow for use in combination with other color fluorescent probes (blue nucleic acid stain and red membrane stain), NBD was chosen as a green-colored fluorescent reporter.

Our conjugation strategy employs the Cu-catalysed azide-alkyne cycloaddition (CuAAC) reaction, which is compatible with the wide range of functionality present on different types of antibiotics, including the free amine and hydroxyl groups present in polymyxin lipopeptides. As mentioned earlier, previous polymyxin-derived fluorescent probes have non-selectively derivatized the multiple free amine groups on the five Dab residues, or modified the exocyclic ‘tail’, with varying effects (often not reported) on activity. Based on our previous structure–activity relationship studies of the polymyxins [57], we identified that position Leu-7 was tolerant to change, and therefore selected this site for fluorophore attachment so that the modification did not affect either the critical free Dab amine groups, or the membrane anchoring exocyclic lipid chain component, both believed to be critical for binding to lipid A [58]. We therefore incorporated an l-azidolysine residue at this position, employing solid phase peptide synthesis with an ‘off-resin’ cyclization of a linear peptide cleaved from the resin (Scheme 1). An unbranched fatty acid tail (octanoyl, C_8_) was used instead of the mixture of natural branched fatty acids found in PMXB, producing the azide-functionalized PMX-P7-N_3_ **6**.

**Scheme 1:**
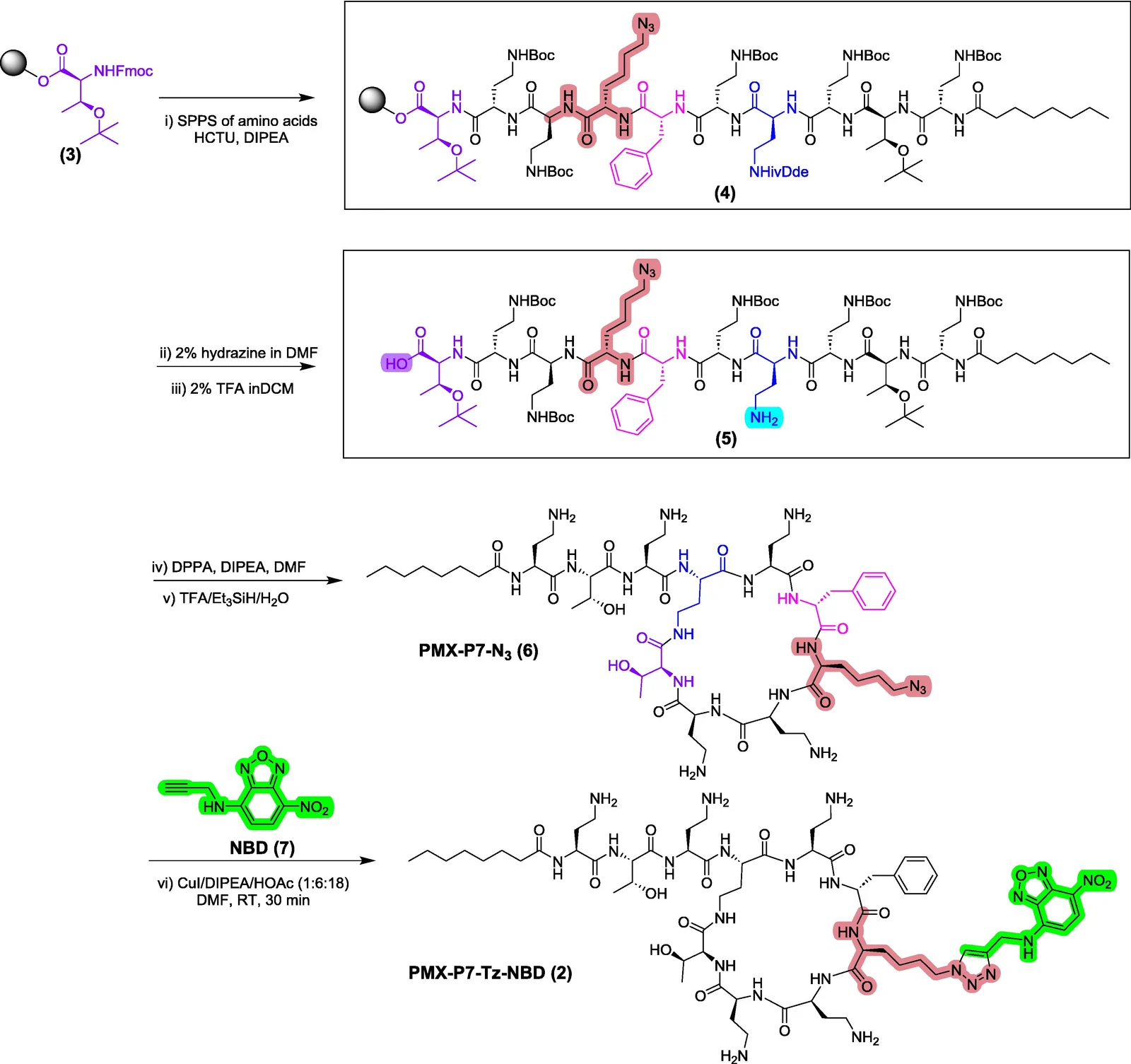
**Synthesis of azido-PMX-P7-N**_**3**_**6 using an ‘off-resin’ cyclization strategy and cycloaddition reaction to generatefluorescent probe PMX-NBD 2.**

The peptide sequence was generated by standard solid-phase peptide synthesis utilizing commercially available Fmoc-Thr(tBu)-2CT resin **3** as a starting point. The linear chain of the azido-PMXB was assembled from the C to N direction by sequential Fmoc deprotection with 30 % piperidine in DMF, followed by HCTU-mediated coupling with amino acids, and with final N-acylation using octanoic acid, to produce resin-linked protected peptide **4**. The ivdDe side chain amine group was selectively cleaved by 2 % hydrazine in DMF, followed by cleavage of the linear peptide from the resin by a solution of 2 % TFA in dichloromethane to obtain a partially protected linear peptide **5**. The linear peptide **5** was then cyclized between the side chain amino group of Dab-4 and carboxy group of Thr-10 using DPPA and DIPEA in DMF, followed by removal of all protecting groups using a solution of TFA/Et_3_SiH/H_2_O (95:1:4) to give azido-PMX-P7-N_3_ **6** (Scheme 1).

The CuAAC conjugation reaction was conducted with NBD-alkyne derivative **7** [53]. Acetic acid was employed as a proton source to accelerate the conversion of the C-Cu bond-containing intermediate (Cu(I) acetylide and 5-cuprated 1,2,3-triazole) in the CuAAC reaction, leading to a reduction in byproduct formation. Optimal conditions for the CuAAC reaction were established to include a room temperature reaction of CuI/DIPEA/HOAc (1:6:18) in DMF (Scheme 1).

### Biological activity

The antimicrobial efficacy of the modified PMXB derivatives was assessed against a panel of Gram-negative bacteria (Table 1). The MIC of PMX-NBD against susceptible *E. coli*, *K. pneumoniae*, *P. aeruginosa*, and *A. baumannii* was very similar to the parent compound, PMXB, with MICs mildly increased against four species, indicating that the site of attachment and use of the small fluorophore NBD were suitable for preparation of lipopeptide probes. Similarly, the azido-PMXB intermediate **6** (PMX-P7-N_3_) retained comparable antimicrobial activity. Encouragingly, both PMX-NBD and PMX-P7-N_3_ lost activity against PMXB non-sensitive strains, supporting the hypothesis that they would retain the same mechanism of action as PMXB. Compared to the known fluorescent dansyl-PMXB lipopeptide probe, which was 4–8 fold less active than PMXB against PMXB-sensitive *K. pneumoniae* ATCC 13883, *P. aeruginosa* ATCC 27853 and *A. baumannii* ATCC 19606 [49], PMX-NBD showed greater potential to reveal the localization and mechanisms of action of lipopeptide antibiotics in Gram-negative bacteria, and potentially mechanisms of nephrotoxicity in mammalian kidney cells. To further verify this, we also performed a time-kill kinetics study to directly compare the bactericidal rate of PMX-NBD versus unmodified PMXB. This experiment serves as a powerful proxy for assessing whether the probe's cellular uptake and lethal action occur on a similar timescale. The results showed that the time-kill curves for PMX-NBD and unmodified PMXB were highly superimposable. Both compounds exhibited a rapid, concentration-dependent killing effect, achieving a > 4-log10 reduction at 2 μg/mL in CFU/mL within 1 h of exposure (Fig. S1). The nearly identical kill curves provide strong evidence that the bacterial “half-life” or the rate of cell death induced by PMX-NBD is statistically indistinguishable from that of the native drug. This implies that the critical processes of initial binding, uptake, and subsequent disruption of the bacterial membrane proceed with similar kinetics.

### Live cell confocal microscope fluorescence imaging of bacteria with PMX-NBD

PMXB serves as a therapeutic agent for drug-resistant Gram-negative bacterial infections. This lipopeptide antibiotic works by adhering to the cell membrane by displacing cations from, and then binding to, the lipid A component of lipopolysaccharide (LPS). This changes the structure of the cell membrane to make it more permeable, and the cell expands and dies due to absorbing too much water from the surrounding environment [59]. A range of additional mechanisms of killing have been proposed, based on intracellular interaction of PMXB once it has penetrated the membrane [60], [61]. Many bacteria, such as *E. coli*, *K. pneumoniae*, *P. aeruginosa* and *A. baumannii* have developed resistance to polymyxin, generally by mechanisms that alter the lipid A structure to reduce its negative charge [62], [63], [64], [65], [66], [67]. To examine the labeling ability of the synthesized fluorescent probe PMX-NBD for bacteria, the four species mentioned above (Fig. 2) were selected and imaged using high-resolution confocal microscope. The bacteria were exposed to the probe PMX-NBD at 2 μg/mL and then incubated at 37 °C for 30 min, followed by co-staining with FM4-64FX (binding to the outer leaf of the plasma membrane) and Hoechst 33342 (cell-permeant nucleic acid stain) (Fig. 2). A range of concentrations and exposure times were explored before arriving at these conditions. The cell membranes of all four strains showed labeling with PMX-NBD, which is consistent with its known mode of action. Significant localized intracellular accumulation in punctuate dots was also observed in all four species; the importance of this observation, potentially indicative of additional mechanisms of action beyond membrane disruption, will be the focus of future studies. Notably, almost no staining of Gram-positive *S. aureus* was observed, confirming the absence of non-selective staining.

**Fig. 2:**
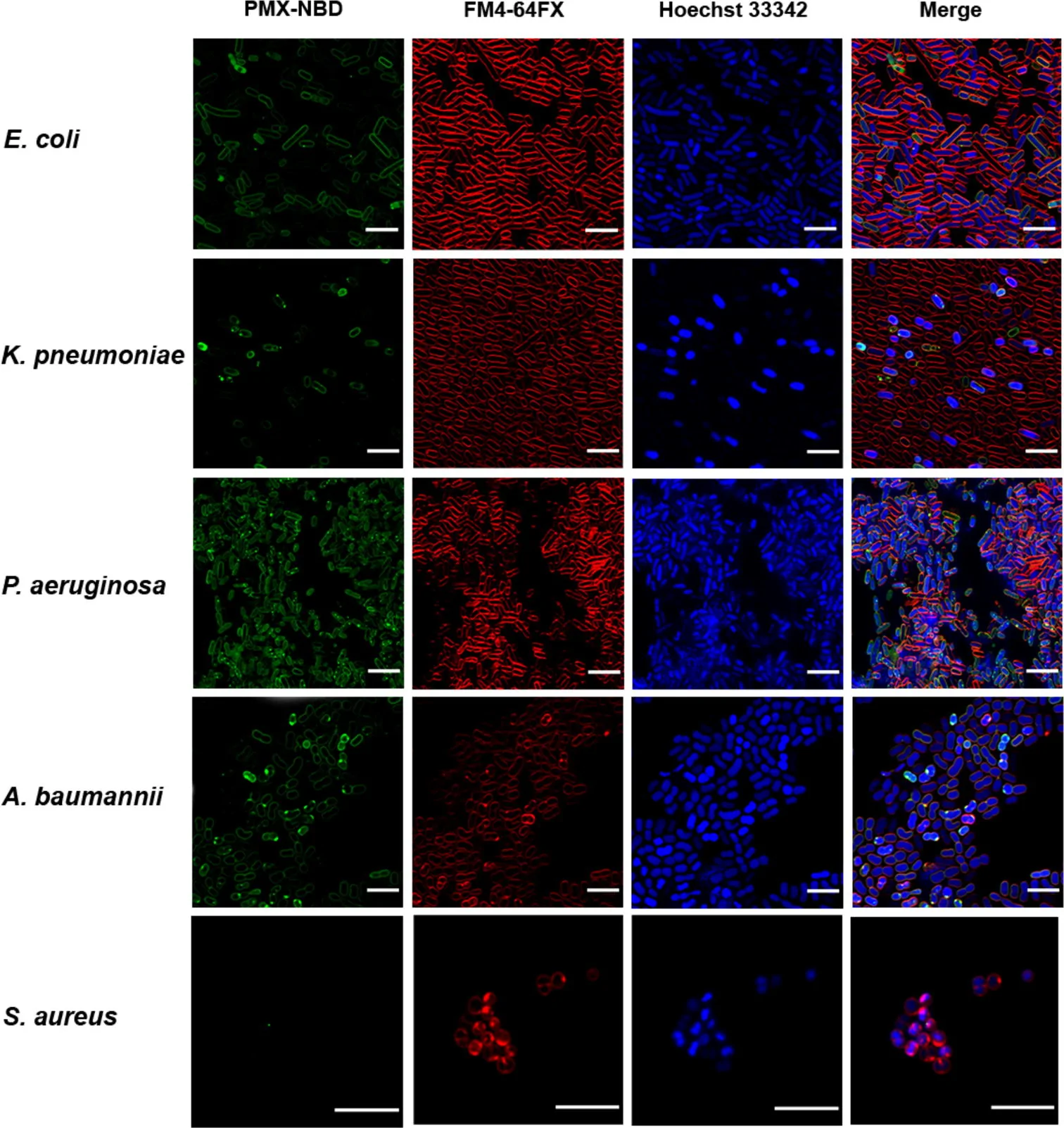
**Live cell confocal microscope fluorescence imaging of*****E. coli*****ATCC 25922,*****K. pneumoniae*****ESBL ATCC 700603,*****P. aeruginosa*****ATCC 27853,*****A. baumannii*****ATCC 19606 and*****S. aureus*****ATCC 25923 treated with PMXB-derived fluorescent probe PMX-NBD 2.** The bacteria were treated with PMX-NBD at 2 μg/mL for 30 min, then co-stained with FM4-64FX and Hoechst 33342. Green: antibiotic-derived fluorescent probes; Red: FM4-64FX (bacterial membrane), Blue: Hoechst 33342 (nucleic acid). Images are representative of *n* = 3 experiments. Scale bar represents 5 μm. (For interpretation of the references to color in this figure legend, the reader is referred to the web version of this article.)

To conduct a more in-depth evaluation and quantification of the specific labeling achieved by PMX-NBD, we analyzed the four Gram-negative and one Gram-positive bacteria co-stained with the probe PMX-NBD (green) and SYTO® 60 (red), a non-specific cell permeable nucleic acid dye. The flow cytometry results showed that SYTO® 60 successfully labeled all 5 bacterial strains while a notable shift in the FITC channel only observed in the Gram-negative strains (95.46 %, 95.10 %, 83.19 %, and 97.87 % labeled for Gram-negative strains, only 10.19 % labeled for Gram-positive strains), confirming the Gram-negative specificity of the probe, which is consistent with the activity of PMXB against these bacteria (Fig. 3).

**Fig. 3:**
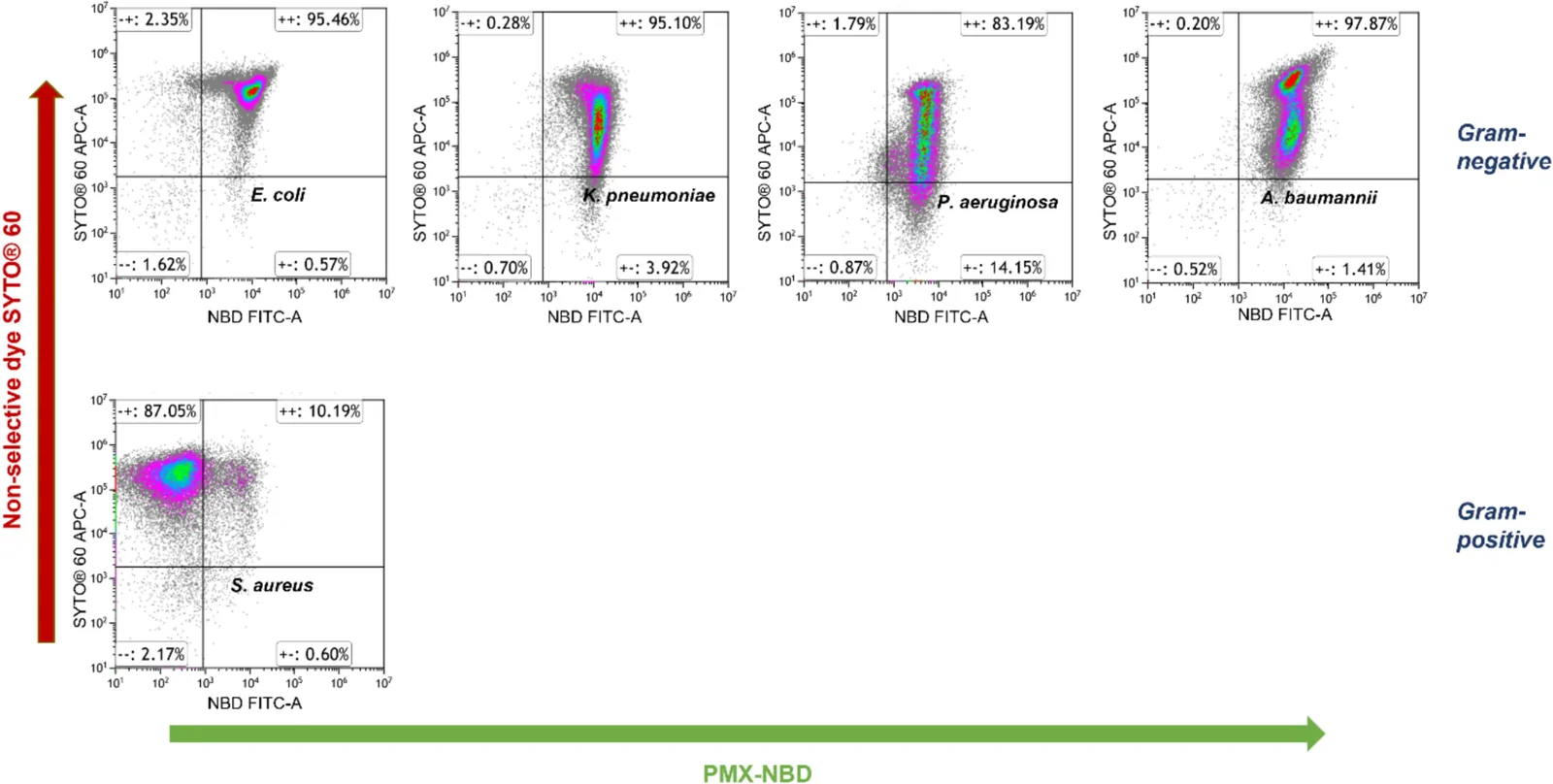
**Flow cytometry analysis of bacteria labeled with PMX-NBD2.** Labeling of bacteria with PMX-NBD and the non-selective cell permeable dye SYTO® 60 in various species using flow cytometry. Bacteria were treated with PMX-NBD at a concentration of 2 μg/mL (37 °C, 30 min) and SYTO® 60 (5 μM, 10 min on ice), then fluorescence intensity was measured using a flow cytometer.

### Determination of PMXB susceptibility with the fluorescent probe PMX-NBD

A rapid fluorescent antibiotic-based flow cytometric antimicrobial susceptibility test method, RAPID_FC_ (Fig. 4), was developed to determine the PMXB susceptibility of a range of Gram-negative bacteria. We chose 49 strains from *E. coli* (*n* = 13), *K. pneumoniae* (*n* = 13), *P. aeruginosa* (*n* = 10), *A. baumannii* (*n* = 12) and *S. aureus* (*n* = 1) with varying levels of PMXB susceptibility, including strains sourced from both the ATCC and CDC Antibiotic Resistant Bank reference collections, as well as clinical isolates provided by a number of international collaborators (Table 2). When subcultures of these isolates reached exponential phase, the bacterial cells were collected and incubated with PMX-NBD at 1 μg/mL and 16 μg/mL for 30 min. Subsequently, the samples were subjected to analysis using a Cytoflex S flow cytometer (Beckman Coulter). Three main parameters were investigated including 1) side scatter (indicating the complexity of the cells); 2) forward scatter (indicating cell size); and 3) the intensity of fluorescence of the cells (indicating labeling of the PMX-NBD probe). To our surprise, initial studies using a fixed probe concentration did not reveal a consistent correlation between absolute fluorescence intensity and polymyxin MIC. However, when the fluorescence was measured at two different concentrations, it became apparent that there were variations in the ***change in fluorescence*** that correlated with the resistance profiles, with a reduced increase in fluorescence at the higher concentration for strains with greater resistance. Therefore, the fluorescence intensity of two concentrations was compared for each tested microbial strain. Susceptibility to PMXB was defined by calculating the difference between the GMean of PMX-NBD fluorescence intensity at 1 μg/mL and 16 μg/mL.

**Fig. 4:**
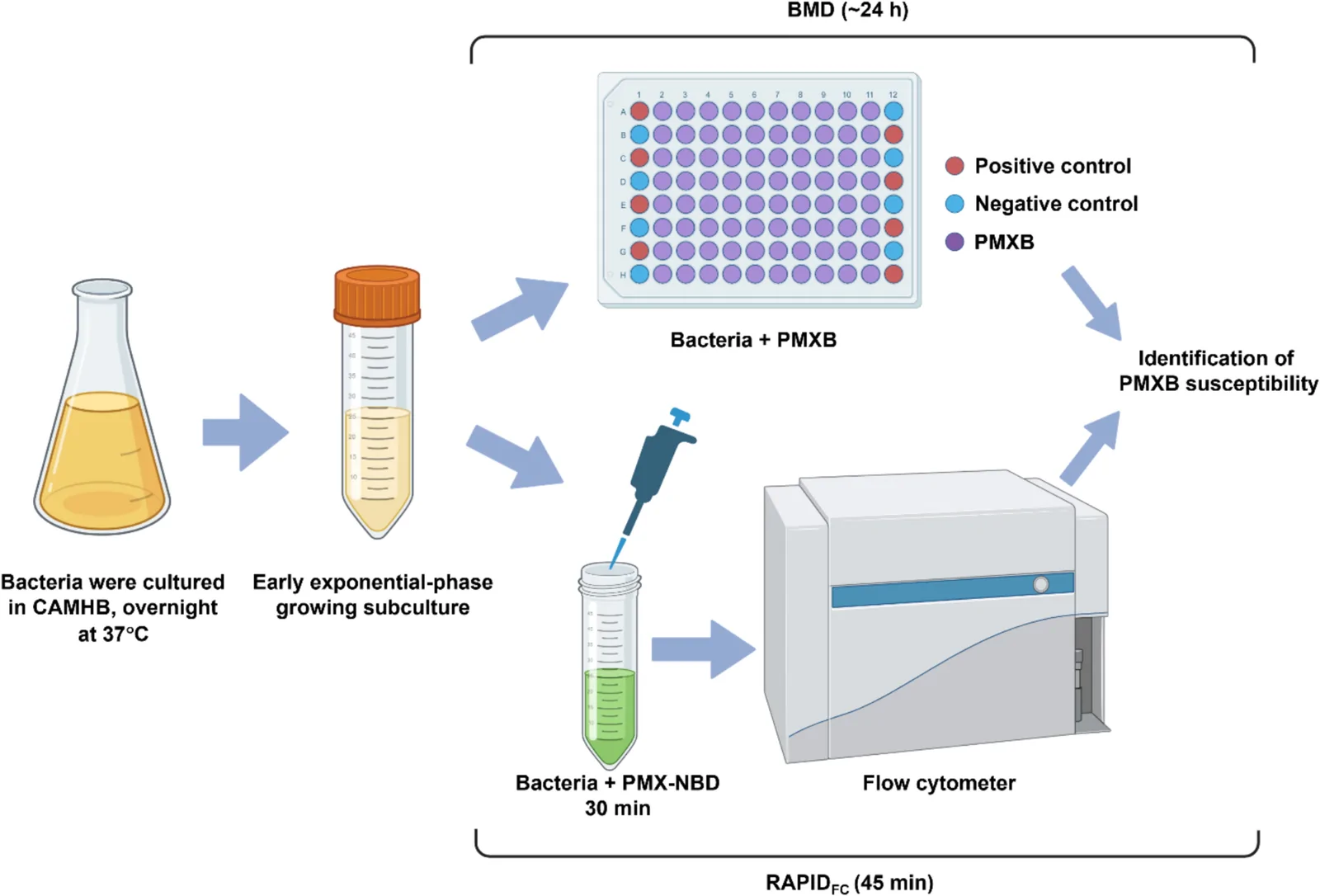
**Workflow of PMX-NBD2based flow cytometry method for PMXB-susceptibility test.** An overnight culture was diluted 40-fold in fresh CAMHB and incubated at 37 °C until mid-log phase, then 1 mL of the culture was collected and incubated with a solution (1 mL) of PMX-NBD at two different concentrations (1, 16 μg /mL) for 30 min at 37 °C with shaking, then analyzed by flow cytometry (Cytoflex S, Beckman Coulter, Inc. 250 S. Kraemer Blvd. Brea, CA 92821 U.S.A.). Figure created with BioRender.com.

According to the GMean values measured at the two concentrations (shown for *E. coli* in Fig. 5A), the increase in mean fluorescence ΔF (normalized to the ‘control’ fluorescence value of *E. coli*) was calculated (Fig. 5B). A PMXB non-susceptible Gram-positive strain, *S. aureus* ATCC 25923, acted as a positive control. In the collection of 13 PMXB-susceptible *E. coli* strains tested, the normalized difference in fluorescence ΔF were all greater than 67, whereas all resistant strains were below 54 (Table 3). Notably, there was still a significant difference between the values determined for the ‘sensitive’ strain with the least variation in fluorescence at the two concentrations, and the ‘resistant’ strain with the greatest variation (p ≤ 0.05 as determined by the *t* test). Decreasing susceptibility of *E. coli* to PMXB led to decreasing change in fluorescence variation at the two concentrations, leading to a significant difference between PMXB-susceptible and PMXB-resistant strains.

**Fig. 5:**
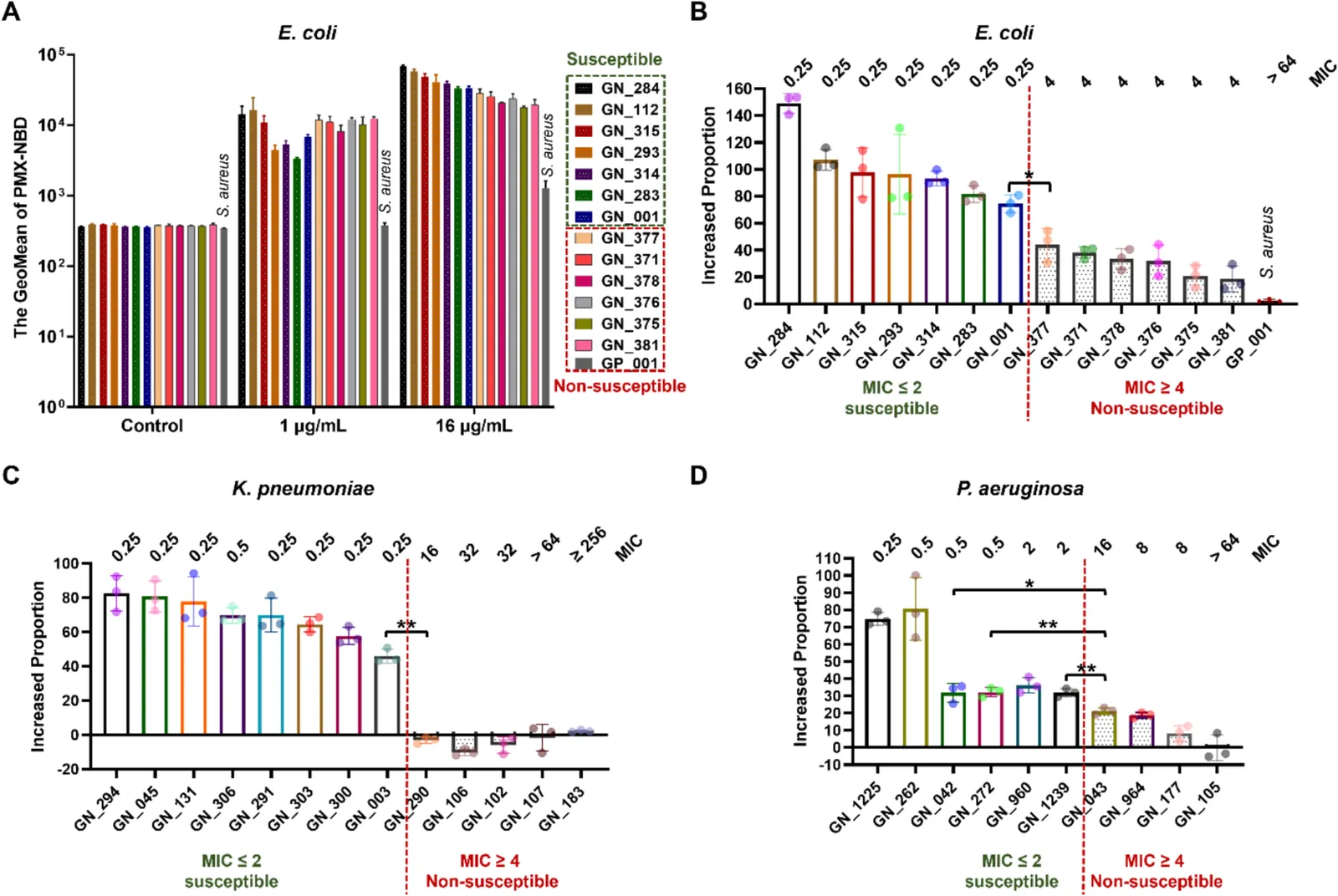
**PMXB-susceptibility test with PMX-NBD 2 using RAPID**_**FC**_**method.** Bacteria were treated with PMX-NBD at 1 μg/mL and 16 μg/mL for 30 min with shaking. Then cells were washed once with HBSS, then the suspended bacteria were diluted in HBSS for flow cytometry measurement. (A) the PMX-NBD GMean of *E. coli* and *S. aureus* after treatment. (B) relative increased fluorescence of *E. coli* or *S. aureus*, calculated by (GMean_16μg/mL_/GMean_control_) − (GMean_1μg/mL_/GMean_control_). (C) increased proportion of *K. pneumoniae* = (GMean_16μg/mL_/GMean_control_) − (GMean_1μg/mL_/GMean_control_). (D) increased proportion of *P. aeruginosa* = (GMean_16μg/mL_/GMean _control_) − (GMean_1μg/mL_/GMean_control_). S: Susceptible to PMXB, MIC ≤ 2 μg/mL; NS: Non-susceptible to PMXB, MIC ≥ 4 μg/mL. The experiment was performed in triplicate. Statistical significance (*P ≤ 0.05, **P ≤ 0.01) is shown between susceptible and non-susceptible strains, as determined by unpaired *t* test. Data reported are the mean ± SD for three independent experiments.

**Table 3:** RAPID_FC_ method to test susceptibility to PMXB.

Species	ΔF (increased proportion of GMean)	Susceptibilities
*E. coli*	> 67	S^a^
*E. coli*	≤ 54	NS^b^
*K. pneumoniae*	> 43	S^a^
*K. pneumoniae*	≤ 3	NS^b^
*P. aeruginosa*	> 26	S^a^
*P. aeruginosa*	≤ 23	NS^b^

a Susceptible to PMXB, MIC ≤ 2 μg/mL.

b Non-susceptible to PMXB, MIC ≥ 4 μg/mL.

We measured the GMean values for 13 isolates of *K. pneumoniae* and 10 isolates of *P. aeruginosa* using the same method (Fig. S2A and S2B) and found the same trends in ΔF for both *K. pneumoniae* (Fig. 5C) and *P. aeruginosa* (Fig. 5D). In a collection of *K. pneumoniae* strains, all ΔF values of the PMXB sensitive strains were over 43, with non-susceptible strains lower than 3. Of note, when susceptible and non-susceptible strains were compared, all non-susceptible strains showed a ΔF value very close to 0, indicating a substantial difference between the two groups of strains in their ability to bind to PMX-NBD. For *P. aeruginosa*, PMXB susceptible strains had ΔF > 26, while the resistant strains were ≤ 23. For both *K. pneumoniae* and *P. aeruginosa*, there was again a statistically significant difference between the ΔF for the ‘sensitive’ strain with the least variation, and the ‘resistant’ strain with the greatest variation.

We also tested *A. baumannii* (Fig. S2C and S2D). In this case there was not a clear distinction between the 5 sensitive and 7 resistant *A. baumannii* isolates as observed for *E. coli*, *K. pneumoniae* and *P. aeruginosa* (Fig. 5 and S2), though these results might be due to an anomalous result for isolates GN_624 and GN_093. It is also possible that a difference in *A. baumannii*’s mechanism of resistance to polymyxin may explain the lack of differentiation. The initial target of PMXB is negative charged LPS, so to protect bacteria from killing by cationic antimicrobial peptides, bacteria themselves employ several strategies, including alterations of LPS [62], operation of an efflux pump [68] and the formation of capsules [69]. In *K. pneumoniae* strains, the phosphate groups of lipid A have been found to contain five times more 4-amino-l-arabinose (l-Ara4N) than the susceptible strain [70]. This cationic substitution of the phosphate groups by l-Ara4N decreases the net negative charge of lipid A to zero, as a result, the modified LPS has a reduced ability to bind to polymyxins, conferring resistance. Similarly, in *P. aeruginosa*, the resistance to polymyxin is related to the addition of l-Ara4N to the phosphate groups within the lipid A moiety of LPS [71]. In polymyxin-resistant *E. coli*, the lipid A structure is commonly modified by 2-aminoethanol and l-Ara4N [72]. Based on this, susceptible strains of these species treated with PMX-NBD at higher concentrations will show more binding of PMX-NBD to the LPS than will non-susceptible strains, resulting in more significant increases in fluorescence. *A. baumannii*, in contrast to Enterobacteriaceae *(E. coli* and *K. pneumoniae)* or Pseudomonadaceae *(P. aeruginosa)*, lacks all genetic machinery necessary for l-Ara4N biosynthesis. *A. baumannii* strains display high-level resistance to polymyxins by modifying the lipid A moiety of LPS with phosphoethanolamine due to mutations in the PmrA/PmrB two-component system, or by completely halting LPS production as a result of mutations or insertional inactivation of genes accountable for lipid A biosynthesis [8], [62]. This difference in mechanism of resistance may explain why this workflow has not clearly differentiated polymyxin resistant and susceptible *A. baumannii*, though it should still lead to reduced binding of the fluorescent probe (Fig. S2C). This variation would be consistent with their mechanism of resistance (i.e. reduction or loss of LPS). We did assess other conditions to optimize PMXB resistance discrimination for *A. baumannii*. To keep the overall measurement time short, we gave priority to optimizing the PMXB-NBD concentration combinations, examining 64 µg/mL *vs* 1 µg/mL; 4 µg/mL *vs* 1 µg/mL; 4 µg/mL *vs* 0.25 µg/mL; 16 µg/mL *vs* 0.25 µg/mL; and 1 µg/mL *vs* 0.25 µg/mL. These adjustments involved a wide range of high, middle and low concentrations (Fig. S3). Flow cytometry confirmed that there was no improvement in the resolution between the susceptible and resistant populations. That is, the results were consistent with those obtained previously. We also explored if fluorescence at only a single concentration rather than the change in fluorescence at two concentrations provided a better correlation with resistance, but again several strains were not consistent (data not shown). Therefore, a 'one-size-fits-all' protocol is not feasible for the specific bacterial species being studied. However, we still hope that in future work, we are able to develop other PMX-NBD-based methods to break through this limitation.

## Conclusion

A new lipopeptide fluorescent probe PMX-NBD was designed and synthesized from azido-lipopeptide building blocks and alkyne fluorophores using the CuAAC reaction. This probe, the first with the fluorophore specifically attached to the cyclic core rather than the linear tail or free amino groups, showed a similar antimicrobial activity profile as the parent antimicrobial, PMXB. Fluorescent imaging of different bacterial species using PMX-NBD showed its ability to label the membrane of at least four Gram-negative bacteria, with nearly no staining of Gram-positive *S. aureus*, and also demonstrated intriguing intracellular localization. With this probe in hand, we developed a RAPID_FC_ method to quickly determine bacterial susceptibility to PMXB using flow cytometry. This method compared the change in fluorescent labeling of the probe at two different probe concentrations, with PMXB-sensitive strains showing a greater increase in fluorescence compared to PMXB-resistant strains (Table 3). The RAPID_FC_ method is easy to implement and only requires 45 min from exponential phase growing culture (30 min of incubation, 8 min for manual handling, 6 min for data acquisition, and 1 min of math calculation), compared to the current standard practice, the broth microdilution method, which demands a minimum of 24 h, with an initial overnight inoculation taking 16–20 h beforehand. Notably, the assay utilizes standard flow cytometry instrumentation, and does not need the specialized equipment required for some other fast antimicrobial susceptibility testing methods [22].

To summarize, the short time frame, straightforward data analysis, and successful application to multiple species make this assay a promising antimicrobial susceptibility test method. Critically, the method required measurement at two different probe concentrations to detect resistance. However, the impact of heteroresistance on the susceptibility testing results with this method still needs to be further discerned. Our findings also reveal that the method's application is not universally uniform across all species, highlighting the necessity of understanding pathogen-specific interactions with molecular probes for broader application. Another limitation of the present study is the constrained sample size for these isolates. The relatively small 'n', coupled with the narrow fluorescence separation observed in *P. aeruginosa*, means that the established cut-off values for these species should be considered provisional. In future studies, we aim to assess considerably more clinical isolates with varying resistance profiles to validate and confidently define robust and universally applicable cut-off values for these pathogens and attempt to achieve a quantitative output by being able to predict the MIC values of PMXB against these strains.

## CRediT authorship contribution statement

M.A.T.B. and B.Z. designed the project. B.Z., W.P., and M.A.T.B. wrote the manuscript with input and review from all authors; M.A.T.B., M.A.C., M.S.B., W.P., R.P., S. Kc., A.G.E., M.A., A.G.G., and B.Z. designed and coordinated experiments and analyzed results, W.P. designed, synthesized, purified and analyzed compounds, B.Z. performed the in vitro microbiological assays, flow cytometry and high-resolution confocal imaging analysis. All authors discussed the results and commented on the manuscript.

## Data and materials availability

All data needed to evaluate the conclusions in the paper are present in the paper and/or the Supplementary Materials.

## Declaration of competing interest

The authors declare that they have no known competing financial interests or personal relationships that could have appeared to influence the work reported in this paper.
