# Supplementary material for: Rapid profiling of polymyxin B susceptibility in Gram-negative bacteria with a novel polymyxin-derived fluorescent probe
Source: J Adv Res. 2026 Jan 10;88:263–74. doi: 10.1016/j.jare.2026.01.019 (PMC13669591; doi:10.1016/j.jare.2026.01.019)
Supplement: Supplementary file 1 [file mmc1.docx]

Supplementary Materials for

**Rapid Profiling of Polymyxin B Susceptibility in Gram-negative Bacteria with A Novel Polymyxin-derived Fluorescent Probe**

Bing Zhang,^1^ Wanida Phetsang,^1^ Sanjaya Kc,^1^ Mark S. Butler,^1^ Ruby Pelingon,^1,2^ Alysha G. Elliott,^1,3^ Maite Amado,^1^ Alejandra Gallardo-Godoy,^1^ Matthew A. Cooper,^1,4^ Mark A.T. Blaskovich^1^*

^1^Centre for Superbug Solutions, Institute for Molecular Bioscience, The University of Queensland, Brisbane, Queensland, Australia.

^2^Current affiliation: School of Chemistry and Molecular Biosciences, The University of Queensland, Brisbane, Queensland, Australia.

^3^Current affiliation: Biointelect Pty Ltd, Brisbane, Queensland, Australia.

^4^Current affiliation: Sitala Bio Ltd, Unit D6, Grain House, Cambridge CB22 5LD, UK.

*Corresponding author. Email: m.blaskovich@uq.edu.au

**This PDF file includes:**

Experimental details for the synthesis and characterization of the PMX-NBD probe.

Table S1 Amino acids and the calculated amounts for synthesis of PMX-P7-N_3_ (6).

Fig. S1 Time-kill curve for *E. coli* ATCC 25922.

Fig. S2 PMXB-susceptibility test with PMX-NBD using RAPID_FC_ method.

Fig. S3 The optimization of PMXB-susceptibility test with PMX-NBD using RAPID_FC_ method for *A. baumannii*.

References [1, 2]

Experimental details for the synthesis and characterization of the PMX-NBD probe

**General**

All materials, unless otherwise noted, were obtained from commercial suppliers and used without further purification. Non-aqueous reactions were conducted under an inert atmosphere of nitrogen. Analytical LCMS was performed on a Shimadzu LCMS 2020 using 0.05% formic acid in water as solvent A and 0.05% formic acid in acetonitrile as solvent B. LCMS conditions (solvent A = H_2_O + 0.05% formic acid, solvent B = acetonitrile + 0.05% formic acid): Column Zorbax Eclipse XDB-Phenyl, 3.0 × 100 mm, 3.5 μm: Flow: 1 mL/min: Gradient timetable: 0.5 min, 5% B; 8.5 min, 100% B; 2.0 min, 100% B; 0.2 min, 5% B. Column chromatography was performed using silica gel 60 (0.063–0.200 mm), 70–230 mesh ASTM. Agilent 1260 Infinity Preparative HPLC with a G1365D multiple wavelength detector set at λ= 210 nm and Grace Reveleris X2 chromatography systems were used for compound purification. Commercially available cartridges were used for MPLC chromatography (Reveleris C18 Reversed-Phase 12 g cartridge), while HPLC purifications used an Agilent Eclipse XDB-Phenyl column 30 × 100 mm, 5 μm particle size. ^1^H (600 MHz) and ^13^C (150 MHz) NMR spectra were obtained using a Bruker Avance-600 spectrometer equipped with a TXI cryoprobe. Chemical shifts are reported relative to the residual solvent signals in parts per million (δ) (D_2_O referenced internally with the HDO resonance at δ_H_ 4.77, and the TFA resonance at δ_C_ 117.978) [1, 2]. High resolution mass spectrometry (HRMS) was performed on a Bruker Micro TOF mass spectrometer (Ultimate 3000) using (+)-ESI calibrated to HCOONa. The MS/MS was performed on SCIEX X500R QTOF.

**Synthesis**

***General procedure A for Fmoc deprotection***

Fmoc protected resin (1.0 g) was treated with a solution of 30% piperidine in DMF (15 mL) and shaken at rt for 15 min. The Fmoc deprotected resin was drained and washed with DMF (× 3), MeOH (× 3) and DCM (× 3). The deprotection step was repeated.

***General procedure B for peptide coupling***

Corresponding amounts of amino acids and other compounds are referred to **Table S1**.

Fmoc deprotected resin (1.0 g), a solution of the corresponding amino acid (2.0 eq.), HCTU (2.0 eq.) and DIPEA (4.0 eq.) in DMF (13.0 mL) was reacted for 1 min and then added to the Fmoc deprotected resin (1.0 g). The resin was shaken for 30 min and then washed with DMF (× 3), MeOH (× 3) and DCM (× 3). The coupling step was repeated. An analytical sample was cleaved with TFA/Et_3_SiH/H_2_O (95:1:4) followed by LCMS analysis to confirm coupling with corresponding amino acid.

***General procedure C for ivDde removal***

A solution of 2% hydrazine hydrate in DMF (15 mL) was added to the reaction vessel containing the corresponding resin **(4)**. The reaction vessel was shaken for 1 h at rt, the solvent was drained and fresh 2% hydrazine hydrate in DMF (15 mL) was added and the vessel shaken for an extra hour at rt. The solvent was drained, washed with DMF (× 3), MeOH (× 3), and DCM (× 3) and dried under vacuum. An analytical sample was cleaved using TFA/Et_3_SiH/H_2_O (95:1:4) followed by LCMS analysis to confirm complete deprotection.

***General procedure D for resin cleavage***

A solution of 2% TFA in DCM (15 mL) was added to the resin and the reaction vessel was shaken for 30 min at rt. The solvent was collected and the resin was washed with DCM (× 3), THF (× 3), and DCM (× 3). The fractions were combined and the solvent was removed under reduced pressure.

***Linear protected azidolipopeptide PMX-P7-N3 5.*** The linear peptide was assembled by SPPS using general procedures **A**-**D** with corresponding amounts of amino acids and other compounds in **Table S1** yielding crude cleaved partially deprotected linear peptide **(5)** (574 mg, 3.08 × 10^-4^ mol.

Table S1.

**Amino acids and the calculated amounts for synthesis of PMX-P7-N_3_ (6)**

| Resin | | Loading (mmol/g) | | Amount (g) | |
| --- | --- | --- | --- | --- | --- |
| Fmoc-Thr(tBu)-2CT | | 0.90 | | 1.0 | |
| **Coupling** | **Amino acid** | **MW** | **eq.** | **mmol** | **Amount (mg)** |
| Step 1 | Fmoc-Dab(Boc)-OH | 440.49 | 2 | 1.80 | 793 |
| Step 2 | Fmoc-Dab(Boc)-OH | 440.49 | 2 | 1.80 | 793 |
| Step 3 | Fmoc-Lys(N_3_)-OH | 394.43 | 2 | 1.80 | 710 |
| Step 4 | Fmoc-D-Phe-OH | 387.43 | 2 | 1.80 | 697 |
| Step 5 | Fmoc-Dab(Boc)-OH | 440.49 | 2 | 1.80 | 793 |
| Step 6 | Fmoc-Dab(ivDde)-OH | 546.65 | 2 | 1.80 | 984 |
| Step 7 | Fmoc-Dab(Boc)-OH | 440.49 | 2 | 1.80 | 793 |
| Step 8 | Fmoc-Thr(tBu)-OH | 397.50 | 2 | 1.80 | 716 |
| Step 9 | Fmoc-Dab(Boc)-OH | 440.49 | 2 | 1.80 | 793 |
| Step 10 | octanoic acid | 144.12 | 2 | 1.80 | 259 |
|  | HCTU | 413.69 | 2 | 1.80 | 745 |
|  | DIPEA | 129.24 | 4 | 3.60 | 627 μL |
|  | DMF |  |  |  | 13 mL |

***Cyclized azidolipopeptide PMX-P7-N3 6.*** To the crude product from the resin cleavage (0.70 mmol) in DMF (130 mL), DIPEA (4.0 eq.) and DPPA (2.0 eq.) were added. The reaction mixture was stirred at 60°C for 16 h. After cooling, the reaction was diluted with EtOAc (150 mL) then washed with water (100 mL, × 2), saturated NaHCO_3_ solution (100 mL), and brine (100 mL). The organic phase was dried over MgSO_4_, filtered and the solvent was removed under reduced pressure. The crude product was treated with TFA/Et_3_SiH/H_2_O (95:1:4) (2.0 mL) for 30 min and the solvent was removed by blowing with N_2_ gas. The compound **6** was purified by preparative HPLC (flow 20 mL/min, mobile phases A = 0.1% TFA in water and B = 0.1% TFA in acetonitrile, gradient 5→100% B over 20 min) and then lyophilised to yield as a white powder (100 mg, 26%).

LCMS: R_t_ = 2.45 min, @ 200 nm, [M + 3H]^3+^ = 411.2, Purity by UV @ 200 nm and ELSD > 95%

^1^H NMR (600 MHz, D_2_O): δ 7.39 (dd, *J* = 7.6, 7.6 Hz, 2H), 7.33 (dd, *J* = 7.4, 7.4 Hz, 1H), 7.26 (d, *J* = 7.1 Hz, 2H), 4.57 (dd, *J* = 8.4, 8.4 Hz, 1H), 4.50-4.46 (m, 3H), 4.34 (d, *J* = 4.3 Hz, 1H), 4.30-4.17 (m, 7H), 3.34-3.29 (m, 1H), 3.25 (dtt, *J* = 6.8, 6.8, 1.9 Hz, 2H), 3.17-3.04 (m, 13H), 2.88-2.84 (m, 1H), 2.79-2.74 (m, 1H), 2.31 (t, *J* = 7.3 Hz, 2H), 2.26-1.82 (m, 12H), 1.73-1.67 (m, 1H), 1.58 (tt, *J* = 7.1, 7.1 Hz, 2H), 1.51-1.42 (m, 3H), 1.27-1.25 (m, 10H), 1.19 (d, *J* = 6.6 Hz, 3H), 1.18 (d, *J* = 6.5 Hz, 3H), 1.18 (o, 1H), 0.99-0.93 (m, 1H), 0.84 (t, *J* = 6.9 Hz, 3H); ^13^C JMOD NMR (150 MHz, D_2_O): δ 178.6, 175.0, 174.0, 173.9, 173.7, 173.5, 173.3, 173.0, 172.4, 172.3, 172.1, 129.8, 129.6, 128.1, 67.7, 66.9, 60.1, 59.5, 56.3, 53.9, 53.4, 52.5, 52.4, 51.8, 51.76, 51.4, 51.1, 37.6, 37.1, 37.0, 36.97, 36.9, 36.8, 36.5, 36.0, 31.6, 31.3, 30.5, 30.3, 29.3, 29.1, 28.91, 28.90, 28.7, 28.5, 28.1, 25.9, 22.8, 22.6, 19.8, 19.4, 14.0; (+)-ESI-HRMS calc for C_55_H_97_N_19_O_13_ [M+2]^2+^: 615.8757, found 615.8751.

*PMX-P7-Tz-NBD;* **(7)**

***CuAAC reaction to generate lipopeptide probe PMX-NBD 2.*** A mixture of azide-lipopeptide PMX-P7-N_3_ **(6)** (35 mg, 2.8 × 10^-5^ mol, 1.0 eq.) and alkyne-NBD **7** (1.5 eq.) were dissolved in DMF (4.0 mL), followed by addition of CuI (10 eq. for NBD), and DIPEA (60 eq. for NBD). After the reaction mixture turned red for the mixture of NBD **(6)**, acetic acid (180 eq. for NBD) was added. The reaction mixture was stirred for 30 min at rt. The crude compound was pre-purified by MPLC over C18 silica gel (Grace Reveleris, A: H_2_O (0.1% TFA), B: ACN (0.1% TFA), 0 → 100% B over 8 min). The compounds **(7)** were repurified by preparative HPLC (flow 20 mL/min, mobile phases A = 0.1% TFA in water and B = 0.1% TFA in acetonitrile, gradient 5→100% B over 20 min) and then lyophilised to yield compound **2** as an orange powder (15 mg, 35%).

LCMS: R_t_ = 2.73 min, @ 200 nm, [M + 4H]^4+^ = 363.2, Purity by UV @ 200 nm and ELSD > 95%

^1^H NMR (600 MHz, D_2_O): δ 8.46 (d, *J* = 8.9 Hz, 1H), 8.11 (s, 1H), 7.01-7.00 (m, 3H), 6.83 (d, *J* = 5.9 Hz, 2H), 6.37 (d, *J* = 9.0 Hz, 1H), 4.50-4.46 (m, 2H), 4.45-4.42 (m, 3H), 4.37 (d, *J* = 8.0 Hz, 1H), 4.34 (d, *J* = 4.3 Hz, 1H), 4.30-4.20 (m, 6H), 4.16 (d, *J* = 4.7 Hz, 1H), 4.08 (dd, *J* = 10.3, 3.7 Hz, 1H), 3.31 (ddd, *J* = 13.9, 7.6, 7.6 Hz, 1H), 3.15-3.03 (m, 11H), 2.84-2.79 (m, 1H), 2.74-2.66 (m, 3H), 2.30 (t, *J* = 7.4 Hz, 2H), 2.27-1.68 (m, 18H), 1.57 (tt, *J* = 7.0, 7.0 Hz, 2H), 1.45-1.39 (m, 1H), 1.26-1.23 (m, 10H), 1.19 (d, *J* = 6.4 Hz, 3H),1.17 (m, 3H), 1.18 (o, 1H), 0.93-0.89 (m, 2H), 0.83 (t, *J* = 7.1 Hz, 3H); ^13^C JMOD NMR (150 MHz, D_2_O): δ 178.6, 174.6, 173.8, 173.7, 173.5, 173.46, 173.3, 173.0, 172.5, 172.4, 172.1, 145.0, 144.3, 139.5, 135.9, 129.3, 129.1, 127.6, 125.3, 67.7, 67.0, 60.1, 59.6, 56.2, 53.7, 53.4, 52.4, 52.3, 51.84, 51.8, 51.2, 50.9, 37.6, 37.2, 37.0, 36.9, 36.86, 36.5, 36.1, 31.6, 31.2, 30.3, 30.2, 29.3, 29.1, 28.94, 28.9, 28.7, 28.66, 25.9, 22.8, 22.6, 19.8, 19.4, 14.0; (+)-ESI-HRMS calc for C_64_H_103_N_23_O_16_ [M+2]^2+^: 724.8977, found 724.8983.

**NMR Spectra for** **lipopeptides PMX-P7-N3 (6) and PMX-P7-Tz-NBD (2)**

^1^H NMR (600 MHz, D_2_O) of PMX-P7-N_3_ **(6)**.

^1^H NMR (600 MHz, D_2_O) of PMX-P7-N_3_ **(6)**.

^1^H NMR (600 MHz, D_2_O) of PMX-P7-N_3_ **(6)**.

^13^C JMOD NMR (150 MHz, D_2_O) of PMX-P7-N_3_ **(6)**.

^1^H NMR (600 MHz, D_2_O) of PMX-P7-Tz-NBD **(2)**.

^1^H NMR (600 MHz, D_2_O) of PMX-P7-Tz-NBD **(2)**.

^1^H NMR (600 MHz, D_2_O) of PMX-P7-Tz-NBD **(2)**.

^13^C JMOD NMR (150 MHz, D_2_O) of PMX-P7-Tz-NBD **(2)**.

**MS-MS Spectra for** **lipopeptides PMX-P7-N3 (6) and PMX-P7-Tz-NBD (2)**

(+)-ESI-TOF-MS/MS of PMX-P7-N_3_ (6).

(+)-ESI-TOF-MS/MS of PMX-P7-Tz-NBD (2).

Fig. S1.

**Time-kill curve for *E. coli* ATCC 25922.** *E. coli* ATCC 25922 was exposed to either PMX-NBD or unmodified PMXB at a range of concentrations for each respective compound. Viable bacterial counts (CFU/mL) were determined at 0, 1, 2, 4, 6, and 24 h. Data reported are the mean ± SD for three independent experiments.

Fig. S2.

**PMXB-susceptibility test with PMX-NBD using RAPID_FC_ method.** Bacteria were treated with PMX-NBD at 1 μg/mL and 16 μg/mL for 30 min with shaking, then were washed once with HBSS and the pellets were resuspended, suspended bacteria solution was then diluted in HBSS for flow cytometry measurement. **(A)** PMX-NBD GMean of *K. pneumoniae* after treatment. S: Susceptible to PMXB, MIC ≤ 2 μg/mL; NS: Non-susceptible to PMXB, MIC ≥ 4 μg/mL. The experiment was performed in triplicate. Data reported are the mean ± SD for three experiments. **(B)** PMX-NBD GMean of *P. aeruginosa* after treatment. S: Susceptible to PMXB, MIC ≤ 2 μg/mL; NS: Non-susceptible to PMXB, MIC ≥ 4 μg/mL. The experiment was performed in triplicate. Data reported are the mean ± SD for three experiments. **(C)** PMX-NBD GMean of *A. baumannii* after treatment. **(D)** increased proportion = (GMean_16μg/mL_/GMean_control_) − (GMean_1μg/mL_/GMean_control_). S: Susceptible to PMXB, MIC ≤ 2 μg/mL; NS: Non-susceptible to PMXB, MIC ≥ 4 μg/mL. Data reported are the mean ± SD.

Fig. S3.

**The optimization of PMXB-susceptibility test with PMX-NBD using RAPID_FC_ method for *A. baumannii*.** Bacteria were treated with PMX-NBD at corresponding concentrations for 30 min with shaking. Cells were washed once with HBSS, then the suspended bacteria were diluted in HBSS for flow cytometry measurement. **(A)** increased proportion of *A. baumannii =* (GMean_64μg/ml_/GMean_control_) − (GMean_1μg/ml_/GMean_control_). **(B)** increased proportion of *A. baumannii =* (GMean_4μg/ml_/GMean_control_) − (GMean_1μg/ml_/GMean_control_). **(C)** increased proportion of *A. baumannii =* (GMean_4μg/ml_/GMean_control_) − (GMean_0.25μg/ml_/GMean_control_). **(D)** increased proportion of *A. baumannii =* (GMean_16μg/ml_/GMean_control_) − (GMean_0.25μg/ml_/GMean_control_). **(E)** increased proportion of *A. baumannii =* (GMean_1μg/ml_/GMean_control_) − (GMean_0.25μg/ml_/GMean_control_).

**References**

1. A. Gallardo-Godoy, C. Muldoon, B. Becker, A. G. Elliott, L. H. Lash, J. X. Huang, M. S. Butler, R. Pelingon, A. M. Kavanagh, S. Ramu, W. Phetsang, M. A. Blaskovich, M. Cooper, Activity and predicted nephrotoxicity of synthetic antibiotics based on polymyxin B. *Journal of Medicinal Chemistry.* **59,** 1068-1077 (2016).

2. B. Becker, M. S. Butler, K. A. Hansford, A. Gallardo-Godoy, A. G. Elliott, J. X. Huang, D. J. Edwards, M. A. Blaskovich, M. A. Cooper, Synthesis of octapeptin C4 and biological profiling against NDM-1 and polymyxin-resistant bacteria. *Bioorganic & Medicinal Chemistry Letters.* **27,** 2407-2409 (2017).
